# Anti-VEGF Drugs Dynamics: Relevance for Clinical Practice

**DOI:** 10.3390/pharmaceutics14020265

**Published:** 2022-01-23

**Authors:** Daniele Veritti, Valentina Sarao, Gianluca Gorni, Paolo Lanzetta

**Affiliations:** 1 Department of Medicine—Ophthalmology, University of Udine, 33100 Udine, Italy; daniele.veritti@uniud.it (D.V.); valentina.sarao@uniud.it (V.S.); 2 Istituto Europeo di Microchirurgia Oculare (IEMO), 33100 Udine, Italy; 3 Department of Mathematics and Computer Science, University of Udine, 33100 Udine, Italy; gianluca.gorni@uniud.it

**Keywords:** age-related macular degeneration, aflibercept, bevacizumab, brolucizumab, burden, intravitreal injections, mathematical model, neovascular, ranibizumab

## Abstract

Background: A drug and disease assessment model was used to evaluate the impact of different treatment regimens on intravitreal ranibizumab, bevacizumab, aflibercept, and brolucizumab concentrations and the proportion of free vascular endothelial growth factor (VEGF) to total VEGF. Methods: A time-dependent mathematical model using Wolfram Mathematica software was used. The pharmacokinetic and pharmacodynamic data for anti-VEGFs were obtained from published reports. The model simulated drug concentration after single and multiple doses of ranibizumab, bevacizumab, aflibercept, and brolucizumab, and it extrapolated time-dependent intraocular free VEGF proportion values. Various fixed treatment regimens (q4, q8, q10, q12) were simulated and evaluated as candidates for clinical utilization. Results: Our mathematical model shows good correlation between intraocular VEGF proportion values and clinical data. Simulations suggest that each anti-VEGF agent would allow for distinct treatment intervals to keep the proportion of free VEGF under threshold levels. Regimens scheduling q8 ranibizumab, q8 bevacizumab, q12 aflibercept, and q10 brolucizumab administration permit to maintain the proportion of unbound VEGF below 0.001%. Conclusions: Fixed q8 ranibizumab, q8 bevacizumab, q12 aflibercept, or q10 brolucizumab regimens may produce adequate intraocular VEGF inhibition.

## 1. Introduction

The development and widespread use of intravitreal endothelial growth factor (VEGF) inhibitors has transformed the management of neovascular age-related macular degeneration (AMD), reducing the number of cases of legal blindness by 50% over the last decade [1,2]. Nowadays, in clinical practice, four VEGF-neutralizing molecules, such as ranibizumab (Lucentis^®^, Genentech, South San Francisco, CA, USA and Novartis Pharma AG, Basel, Switzerland) aflibercept (Eylea^®^, Regeneron Pharmaceuticals, Tarrytown, NY, USA and Bayer HealthCare, Berlin, Germany), brolucizumab (Beovu^®^, Genentech, South San Francisco, CA, USA and Novartis Pharma AG, Basel, Switzerland), and bevacizumab (Avastin^®^, Genentech, S. San Francisco, CA, USA/Roche, Basel, Switzerland) have been effectively used to treat neovascular AMD, although only the first three have received United States Food and Drug Administration and European Medicines Agency approval for intravitreal use [3,4]. A considerable amount of data have been accumulated regarding the pharmacokinetic characteristics of anti-VEGF drugs that may guide dosing frequency and optimize clinical efficacy [5,6,7,8,9,10]. Unfortunately, according to their intraocular pharmacokinetic properties, these drugs have relatively short half-lives, thereby requiring frequent injections to maintain their efficacy. To mitigate the treatment burden associated with intravitreal dosing of anti-VEGF drugs, research has focused on identifying agents with molecular details, pharmacokinetic (PK) and pharmacodynamic (PD) properties that promote optimal dosing regimens (with reduced frequency of injection and treatment burden) by maintaining VEGF inhibition and optimizing overall treatment outcomes [11,12,13]. PK principles are important in optimizing dosing regimens. Knowing that the VEGF suppression within ocular tissue is not directly measurable in vivo, the PK data of molecules that bind VEGF have been determined in humans and preclinical animal species by direct analysis of drug levels in the aqueous or vitreous humor or by indirect modeling of serum drug levels [14,15,16,17]. However, substantial gaps in our knowledge regarding clinically applicable pharmacokinetics remain. Mathematical models are often employed to identify, characterize, or analyze PK and PK/PD relationships, and model-informed drug development is increasingly becoming an integral part of drug research [18,19,20]. 

The purposes of the present study are as follows:

- Collect the most relevant PK and PD aspects of the intravitreal administration of ranibizumab, bevacizumab, aflibercept, and brolucizumab in human eyes. The use of modeling and simulations is aimed at investigating the vitreous PK of anti-VEGF drugs and at quantitatively analyzing the impact of molar drug load, vitreous half-life, and binding affinity on the duration of drug action;
- Identify a clinically relevant threshold level for free VEGF and elaborate treatment strategies characterized by sub-threshold free VEGF levels. Such a treatment regimen should balance clinical benefits and reduce burden when applied in real-life settings, being thereafter clinically appealing and deserving further clinical validation in phase IV studies.

## 2. Materials and Methods

### 2.1. Systematic Literature Review

A search strategy was developed to identify PK and PD data for four intravitreal anti-VEGF agents (ranibizumab, bevacizumab, aflibercept, and brolucizumab) in human eyes. Outcomes of the most relevant pivotal trials in the treatment of neovascular AMD for each drug were also collected. Clinical trials were identified for and retrieved from major electronic databases, including EMBASE, PubMed, and Cochrane. The last research of the databases was conducted in October 2021 by two reviewers (DV and VS). The research methodology was focused on the combination of medical subject headings and the keywords: “age-related macular degeneration”, “choroidal neovascularization”, “anti-VEGF”, “AMD”, “CNV”, “aflibercept”, “bevacizumab”, “ranibizumab”, and “brolucizumab”. The review was restricted to peer-reviewed clinical articles, publications in English, and those issued until October 2021.

### 2.2. Mathematical Model

A drug and disease assessment mathematical model for the treatment of wet AMD was developed to compare the time-related intravitreal concentrations of anti-VEGF drugs and the proportion of VEGF not bound to the drug. The intravitreal half-lives and kinetic binding parameters (VEGF-A165) are summarized in Table 1. 

Mathematica software version 12.0 (Wolfram Research, Inc., Champaign, IL, USA) was used to generate the profiles representing the intravitreal concentration of the drug and the free VEGF proportion following a series of intravitreal injections. Various regimens were simulated (e.g., monthly (q4), bimonthly (q8), every 10 weeks (q10), quarterly (q12)).

The differential equation used for the present model was:

(1) $$x\left(t\right)=-\frac{log\left(2\right)}{\tau }x\left(t\right)+\frac{d}{4}\overset{n-1}{\underset{k=0}{\sum }}\delta \left(t-k\tau \right)\;\;$$

where

- *t* is time;
- *x*(*t*) is the drug concentration at time t;
- d is the dose;
- *δ* is the Dirac delta function (impulsive jolt);
- *τ* is the interval between injections;
- *n* is the total number of injections.

The equation used to describe the proportion of VEGF not bound to the drug was:

(2) $$\mathrm{Kd}=\frac{{\left[A\right]}^{x}x{\left[B\right]}^{y}}{\left[\mathrm{AxBy}\right]}\;$$

where

- Kd is the dissociation constant; and
- [A], [B], and [AxBy] are the molar concentrations of the anti-VEGF agent, VEGF, and the complex, respectively.

A linear, one-compartment model was used to describe drug elimination from the vitreous. Molar drug concentrations were calculated based on drug dose. Molecular weight and PK/PD parameters used as a basis for simulations are derived from the published literature. Initial vitreous molar drug concentrations were based on a vitreous volume of 4 mL and labeled drug dose. Flip-flop PK relationships for each drug were assumed, with kinetics dominated by the slower process. An extensive literature review was performed in order to identify half-lives to use in our model. For each drug, the authors selected the relevant publications reporting systemic, aqueous, or vitreous half-lives. The highest priority on the selection of the value was given to human studies over primates or other animal models and to studies that directly measured intraocular drug concentration over serum measurements. For ranibizumab, we selected a value of 7.19 days. The estimation of Krohne et al. is derived from aqueous sampling in non-vitrectomized eyes after a 0.5 mg ranibizumab intravitreal administration [21]. This estimate is consistent with intravitreal aflibercept VEGF suppression being 2 times longer than ranibizumab. For bevacizumab, we selected a value of 9.82 days. In human non-vitrectomized eyes, the aqueous half-life parameter after the intravitreal administration of 1.5 mg of bevacizumab was reported being 9.82 days by Krohne et al. [22]. A value of 9.1 days was selected for aflibercept, as the work from Do et al. is the only publication that reports the aqueous half-life of this drug in neovascular AMD patients [23]. A value of 5.1 days was selected for brolucizumab. No intraocular half-life studies are available for brolucizumab. Under flip-flop PK assumption, the vitreous half-life is deducible from the serum half-life after intravitreal administration. The estimation of Holtz et al. was selected, as it provides the least conservative mean estimate of half-life using serum concentration versus time after the intravitreal administration of brolucizumab [24].

## 3. Results

### 3.1. Systematic Literature Review

#### 3.1.1. Ranibizumab

Ranibizumab is a fragment of a recombinant, humanized, monoclonal antibody Fab (48 kDa) that binds to and inhibits all the biologically active forms of VEGF-A. It is produced in the *E. Coli* expression system (not glycosylated) and undergoes an affinity maturation process [3]. 

Ranibizumab was approved by the United States Food and Drug Administration for treating neovascular AMD in 2006 as a consequence of the results of phase III MARINA and ANCHOR trials. Despite its encouraging clinical study outcomes, real-world studies have been challenged to replicate the vision gain that patients experienced in the clinical studies of ranibizumab over the long term. The monthly administration of ranibizumab allows a significant improvement of the visual acuity to the detriment of a remarkable burden on patients, caregivers, clinicians, and healthcare systems [25,26]. Some studies have been conducted to investigate if a less frequent dosage after the initial loading phase would maintain the good visual outcomes of a monthly fixed regimen. A quarterly dosing after three initial monthly injections in the PIER and SAILOR studies showed a steady decline in visual acuity after the initial gain obtained during the loading phase (three initial monthly injections) [27].

##### Pharmacokinetic Studies

On the basis of data from hundreds of samples collected during the registrational phase 3 trial MARINA and several smaller studies, a population PK model was conducted with the intention to illustrate ranibizumab PK in patients with AMD [10,25,28,29]. The authors reported that the systemic concentration–time data for ranibizumab were accurately defined by a one-compartment model with first-order absorption into and first-order elimination from the systemic circulation [10]. Vitreous elimination half-life was rated to be roughly 9 days, and the intrinsic systemic elimination half-life was counted to be nearly 2 h. After an intravitreal administration, ranibizumab leaves slowly into the systemic bloodstream, resulting in an apparent serum half-life of 9 days [10]. The systemic-to-vitreous exposure ratio was estimated to be 1:90,000 [10]. With monthly and quarterly intravitreal regimens, the serum concentrations of ranibizumab at steady state for 0.5 mg dose levels were calculated to be below the range needed to inhibit VEGF-A-induced endothelial cell proliferation in vitro by 50% at all times [29]. By directly measuring aqueous half-life, the vitreous half-life of ranibizumab was 7.2 days after a single 0.5 mg intravitreal injection [21]. Data from vitreous samples on the levels of ranibizumab are very limited because of the difficulty of sampling in vivo in those patients who do not need vitrectomy. No studies to date have found any conclusive evidence of vitreous levels of ranibizumab after intravitreal injection in AMD human eyes.

#### 3.1.2. Bevacizumab

Bevacizumab is a full-length, humanized, recombinant monoclonal antibody directed to all isoforms of VEGFA. Its molecular weight is 149 kDa, and it was conceived for intravenous infusion by recombinant DNA technology with a similar affinity to the original murine antibody [3]. Initially licensed for the treatment of colon cancer, intravitreal 1.25 mg bevacizumab has been used off-label since 2005 as a therapy for neovascular AMD. It is cheaper than many other anti-VEGF agents, as each vial can be fractionated into smaller doses for intraocular use. Pharmacodynamics studies have demonstrated that the binding site of bevacizumab has a 14-fold lower binding affinity than ranibizumab for VEGF-A [30]. However, several studies established intravitreal bevacizumab’s efficacy and safety, although the best evidence comes from the Comparison of Age-related Macular Degeneration Treatment Trials (CATT). The aim of this prospective, randomized study was to evaluate the relative efficacy of ranibizumab and bevacizumab and to determine whether an as-needed (pro-re-nata, PRN) regimen would compromise long-term visual acuity, as compared with a monthly regimen. Twelve-month data reported that monthly bevacizumab was non-inferior to monthly ranibizumab (8.0-letter gain versus 8.5-letter gain, respectively), PRN bevacizumab was non-inferior to PRN ranibizumab (5.9-letter gain versus 6.8-letter gain, respectively), and PRN ranibizumab was not inferior to monthly ranibizumab. The comparison between PRN bevacizumab and monthly ranibizumab and between PRN bevacizumab compared to monthly bevacizumab was inconclusive (did not meet non-inferiority). The percentage of patients who gained more than 15 letters was 34.2% for monthly ranibizumab and 31.3% for monthly bevacizumab during the first 52 weeks of follow up and was respectively 24.9% and 28.0% in the PRN treatments regimens. Patients recruited in the monthly ranibizumab and bevacizumab treatment regimens received a mean of 11.7 and 11.9 injections, respectively. The number of treatments for the PRN bevacizumab arm was 7.7 in comparison to 6.9 for the ranibizumab as-needed arm [31].

##### Pharmacokinetic Studies

The off-label use of bevacizumab in ophthalmology is accompanied by very limited information about its intravitreal pharmacokinetics. 

Previous studies indicated that intravitreal bevacizumab followed a two-compartment model in non-vitrectomized human eyes with initial and terminal half-lives of 0.5 and 6.7 days, respectively. The peak concentration (165 µg/mL) was reached on the second day after intravitreal dosing [15]. These parameters were verified by another study reporting that the clearance of intravitreal bevacizumab was calculated to have a half-life that ranged between 2.5 and 7.3 days, with a mean of 4.9 days [32]. In previously vitrectomized eyes, the vitreal concentration of bevacizumab following intravitreal administration resulted in a calculated half-life of 0.66 days. This value is significantly shorter than that found in non-vitrectomized eyes, and the reason for this is that vitreous acts as a reservoir for the drug, and its absence leads to a more rapid clearance from the eye [32]. Some studies reported a systemic half-life of 18.7 days [30] after three intravitreal monthly injections. Its systemic exposure was found to be greater than that of ranibizumab or aflibercept, with a mean serum concentration of 1.58 nM, which is higher than the estimated inhibitory concentration for VEGF factor (IC50 = 0.668 nM) [14]. This may result in an increased risk of systemic adverse events in comparison with the other anti-VEGFs tested [33,34,35]. Some studies investigated the aqueous concentrations of bevacizumab after a single intravitreal injection. In human non-vitrectomized eyes, the aqueous half-life of 1.5 mg of bevacizumab when injected intravitreally was 9.82 days. The concentration in aqueous humor peaked on the first day after treatment with a mean concentration (Cmax) of 33.3 µg/mL (range, 16.6 to 42.5 microg/mL) and decreased thereafter in a mono-exponential fashion [22]. In the anterior chamber of untreated fellow eyes, no significant levels of unbound bevacizumab were detected [36].

#### 3.1.3. Aflibercept

Aflibercept is a 115 KDa fully human recombinant fusion protein comprised of the extracellular domains 2 and 3 from VEGFR1 and VEGFR2, and it is fused to the Fc region of human IgG1. Aflibercept was designed by linking the amino acid sequences from the principal binding domains of the two human VEGF receptors into a human IgG-1 Fc framework. Aflibercept shows a great affinity for VEGF A, B, and placental growth factor [3]. The three-dimensional structure of aflibercept enables it to simultaneously bind both sides of the VEGF dimer in a “two-fisted grasp”. This results in a higher binding affinity for VEGF165 (kD = 0.45 pM) compared to ranibizumab (kD = 46–172 pM) and bevacizumab (kD = 58–1100 pM) [6].

Aflibercept was approved by the United States Food and Drug Administration for treating the neovascular AMD in 2011. Following completion of the phase III VIEW trials, 2 mg aflibercept was initially approved with a fixed bimonthly regimen. VIEW 1 and VIEW 2 were two large, phase III, clinical trials in which 2457 patients with naïve neovascular AMD were randomized to receive one of four treatments: 0.5 mg ranibizumab monthly, 0.5 mg aflibercept monthly, 2 mg aflibercept monthly, or 2 mg aflibercept dosed every 2 months after an initial loading dose of 3 monthly injections [37]. In these non-inferiority trials, visual improvement was +9.3 letters in the 2 mg monthly aflibercept arm, +8.7 letters in the 0.5 mg monthly ranibizumab arm, +8.4 letters in the 2 mg aflibercept every 2 months arms, and +8.3 letters in the 0.5 mg monthly aflibercept. The percentage of patients who gained 15 ETDRS letters or more from baseline were 32.4%, 33.4%, 29.8%, and 31.0% for the ranibizumab monthly, 2 mg aflibercept monthly, 0.5 mg aflibercept monthly, and 2 mg aflibercept every 2 months groups, respectively. All dosages and treatment strategies of aflibercept were non-inferior to monthly ranibizumab [37]. 

##### Pharmacokinetic Studies

The data on the PK of aflibercept are sporadic and mostly refer to animal models in comparison with the other two anti-VEGF drugs. In initial PK studies, Stewart et al. used a first-order decay model to plot the VEGF-Trap activity for different doses in comparison to ranibizumab. Their study reported that at 83 days after a single intravitreal injection of 2 mg of VEGF-Trap, its VEGF binding activity was comparable to that of ranibizumab at 30 days, suggesting a longer duration of action [13]. In another study, based on a population PK model, Stewart et al. estimated a vitreous half-life of aflibercept to be 7.13 days in human eyes [19]. A recent prospective, interventional case series of five eyes with neovascular AMD was conducted to assess the half-life of aflibercept in aqueous humor after a single intravitreal injection. The authors showed that median peak concentration (Cmax) of unbound aflibercept in the aqueous was 122 mg/L. The mean half-life of free aflibercept was 9.1 days in the eye. In plasma, the concentrations of free aflibercept were low and transient, reaching undetectable levels during the first week after injection, and they were untraceable in all patients beyond 7 days [23]. A study by Avery et al. demonstrated differences in systemic PK and PD among the three main intravitreal anti-VEGF drugs in a population of patients affected by neovascular AMD. In this study, bevacizumab and aflibercept caused a rapid suppression of plasma free VEGF soon after a single intravitreal injection, while free VEGF levels remained largely unchanged among patients who received ranibizumab. Additionally, unlike ranibizumab, bevacizumab and aflibercept cumulated in the blood after the third dose. Although by no means conclusive, these data may provide biological plausibility for potential discrepancies in systemic safety risk. In accordance with the geometric mean ratio of Cmax, Cmin, and area under the curve (AUC) respectively, following the first dose, systemic exposure to aflibercept was 5-fold, 37-fold, and 9-fold higher than ranibizumab, whereas that of bevacizumab was 9-fold, 310-fold, and 35-fold higher than ranibizumab [38]. Another study published more recently by Avery et al. [30] evaluated the systemic PK and PD of ranibizumab, aflibercept, and bevacizumab in patients with AMD. The results endorsed earlier findings, with no systemic accumulation of ranibizumab, and higher plasma concentrations with both aflibercept and bevacizumab. Treatment with aflibercept resulted in the greatest reduction in plasma free VEGF compared to baseline, whereas ranibizumab had the smallest effect on plasma unbound VEGF.

#### 3.1.4. Brolucizumab

Brolucizumab is the newest commercially available anti-VEGF drug. It was approved by the FDA in October 2019 for the treatment of neovascular AMD. Brolucizumab is a single-chain antibody fragment that targets all isoforms of VEGF-A. This agent was designed and developed to decrease the treatment burden, as its low molecular weight of 26 kDa and high solubility allow for the delivering of increased molar equivalents in comparison to other anti-VEGF agents, which in turn may permit longer intervals between treatments. The absence of the crystallizable fragment and its small size allow improved bioavailability with enhanced tissue penetration and more sustained effect than full-size antibodies [4,39]. Thanks to its small molecular size, this drug can be concentrated in a smaller volume, allowing it to supply 6 mg of brolucizumab in as little as 50 μL for intravitreal injection, meaning 11 times higher than aflibercept. The HAWK and HARRIER trials were 2-year, double-masked, multicenter phase III studies exploring the safety and efficacy of brolucizumab versus aflibercept in treatment-naïve patients with neovascular AMD. These studies found that one-half of participants could be maintained on a 12-week dosing of brolucizumab with non-inferiority to eight-week dosing of aflibercept [40]. These studies also recorded that brolucizumab had greater efficacy in neovascular AMD in terms of resolution of subretinal and intraretinal fluid relative to aflibercept. A least squares mean change in visual acuity from baseline to week 48 for brolucizumab was non-inferior to aflibercept in both studies (*p* < 0.001, both studies). At week 48, in the HAWK study, changes in BCVA (standard error) for brolucizumab 3 mg, brolucizumab 6 mg, and aflibercept 2 mg were on average +6.1 (0.69), +6.6 (0.71), and +6.8 (0.71) ETDRS letters, respectively. At week 48, in the HARRIER study, changes in BCVA (standard error) for brolucizumab 6 mg and aflibercept 2 mg were on average +6.9 (0.61) and +7.6 (0.61) letters, respectively. Mean changes in BCVA over weeks 36–48 were also reported as non-inferior in both studies (*p* < 0.001, both studies). Significantly fewer eyes had IRF or SRF in the brolucizumab group versus aflibercept at week 16 (HAWK 3 mg, 42% vs. 52%; *p* < 0.003 and 6 mg, 34% vs. 52%; *p* < 0.001 and HARRIER 6 mg, 29% vs. 45%; *p* < 0.001) and week 48 (HAWK 3 mg, 34% vs. 45%; *p* = 0.002 and 6 mg, 31% vs. 45%; *p* < 0.001 and HARRIER 6 mg, 26% vs. 44%; *p* < 0.001). In addition, in both HAWK and HARRIER at weeks 16 and 48, fewer patients on brolucizumab 6 mg had sub-RPE fluid compared with aflibercept [40].

##### Pharmacokinetic Studies

In phase I and phase II studies (RTH258-C-10-083 and RTH258-E003, respectively) and in few phase III studies that enrolled patients with neovascular AMD, PK sampling was conducted to record the systemic exposure and define PK parameters after intravitreal use [24,39,40,41]. Following intravitreal administration, the systemic levels were low but quantifiable for up to four weeks post-injection in most subjects. The peak serum concentration was low and generally noted within the first day following administration (i.e., at either 6 or 24 h after injection). After that, the brolucizumab concentration declined in a mono-exponential fashion with a harmonic mean half-life of approximately 4.5 days [39,41]. In the work by Holz et al., the mean vitreous half-life was estimated to be 5.1 days [24]. Pre-existing ADA status was demonstrated to have no impact on the half-life of free brolucizumab. PK data from phase I and II and pivotal phase III studies in patients with neovascular AMD suggest a low systemic exposure following intravitreal administration of brolucizumab [39,40,41]. In clinical practice, brolucizumab is to be administered monthly for the first three initial injections and thereafter every 8 or 12 weeks, following functional and morphologic criteria.

### 3.2. Mathematical Model

**Figure 1:**
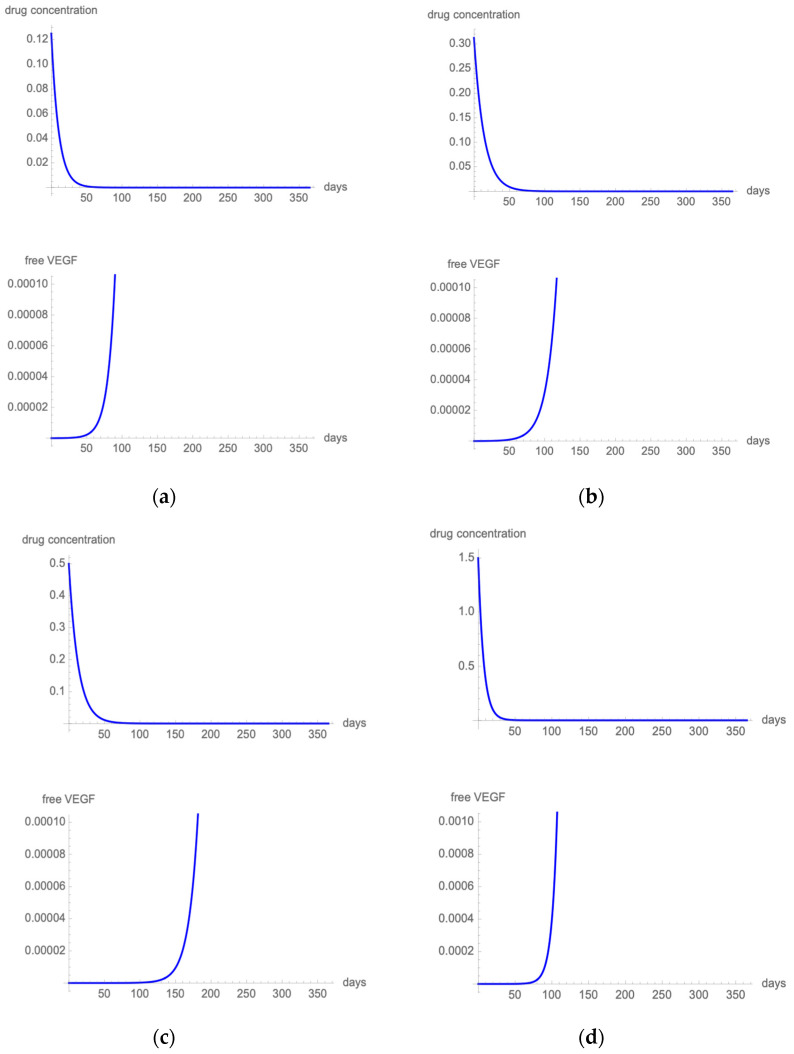
Figure 1 illustrates single-dose PK simulations after intravitreal dosing of 0.5 mg ranibizumab (**a**), 1.25 mg bevacizumab (**b**), 2 mg aflibercept (**c**), and 6 mg brolucizumab (**d**). In the upper section of the figure, a linear, one-compartment time-dependent model describes drug elimination from the vitreous. In the lower part of the figure, a PK-PD model describes the proportion of VEGF not bound to the anti-VEGF drug at a specific timepoint.

The pharmacokinetics data, kinetic binding parameters, and molecular masses of anti-VEGF agents are shown in Table 1. Figure 1 (ranibizumab (Figure 1a); bevacizumab (Figure 1b); aflibercept (Figure 1c); and brolucizumab (Figure 1d)) shows the effect of one intravitreal injection in terms of drug concentration over time and proportion of free-VEGF over time for the four anti-VEGF agents considered in the present work.

#### 3.2.1. Regimen Optimization during the Maintenance Phase 

##### Ranibizumab

**Figure 2:**
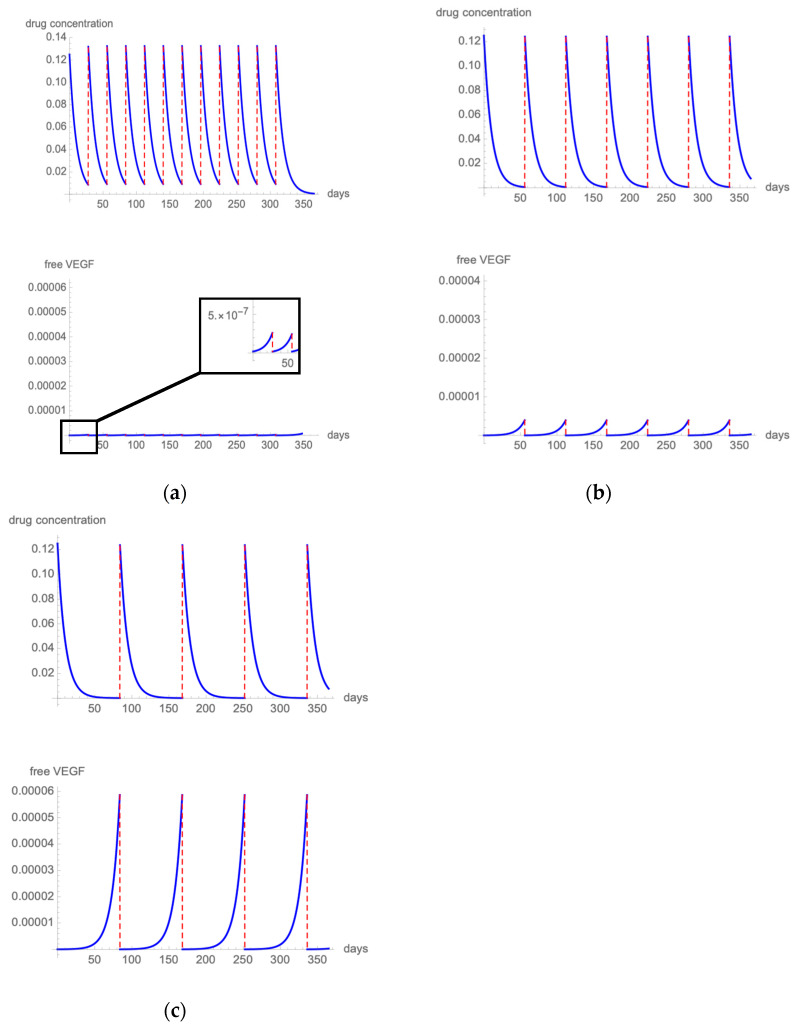
Figure 2 illustrates the intravitreal drug concentration and free VEGF proportion in a patient treated with q4 (**a**), q8 (**b**), or q12 (**c**) 0.5 mg ranibizumab injections.

In the fixed monthly 0.5 mg ranibizumab regimen, which was evaluated in the MARINA, ANCHOR, and CATT trials [25,26,31], the highest free VEGF levels calculated with our model were below 0.0001% of total VEGF (Figure 2a), which means that only one molecule of VEGF over 1,000,000 is free to bind the cellular VEGF receptor. Figure 2b presents the results of the modeling drug kinetics and VEGF inhibition when 0.5 mg ranibizumab is administered bimonthly. VEGF was strongly inhibited, with free VEGF level always below 0.001% of total VEGF. Following the quarterly administration of 0.5 mg ranibizumab (such as in the PIER trial [27]), the highest free VEGF levels reached 0.006% of total VEGF (Figure 2c). 

##### Bevacizumab

**Figure 3:**
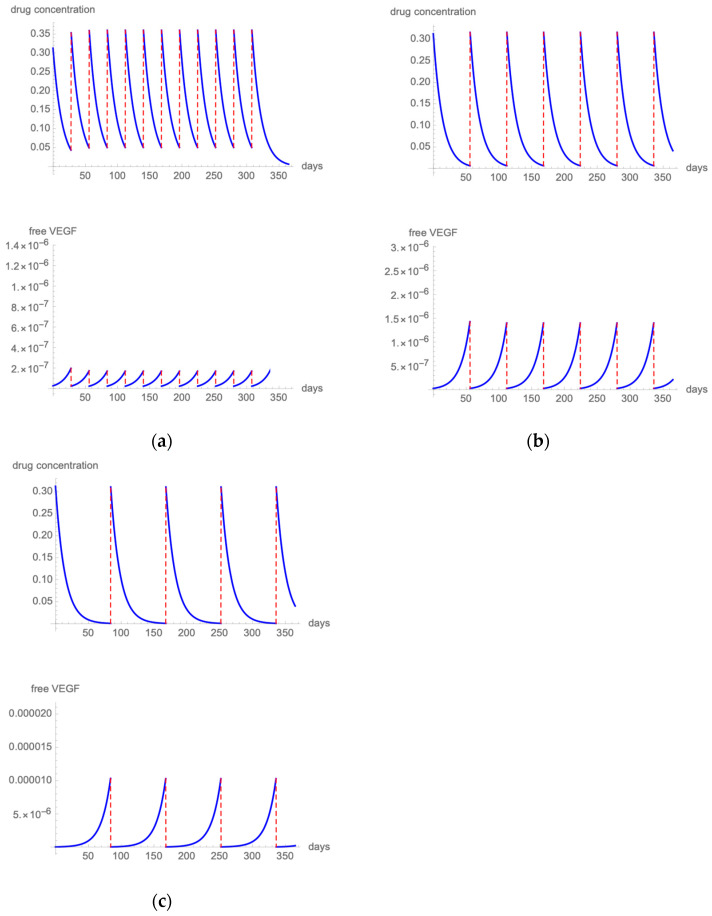
Figure 3 illustrates the intravitreal drug concentration and free VEGF proportion in a patient treated with q4 (**a**), q8 (**b**), or q12 (**c**) 1.25 mg bevacizumab injections.

In the fixed monthly 1.25 mg bevacizumab regimen, which was evaluated in the CATT trial [31], the highest free VEGF levels calculated with our model were below 0.0001% of total VEGF (Figure 3a). In the fixed bimonthly 1.25 mg simulation, the highest free VEGF levels remained well below 0.001% of total VEGF (Figure 3b). On the contrary, extending the treatment interval to a q12 regimen produces spikes of free VEGF that exceed 0.001% (Figure 3c).

##### Aflibercept

**Figure 4:**
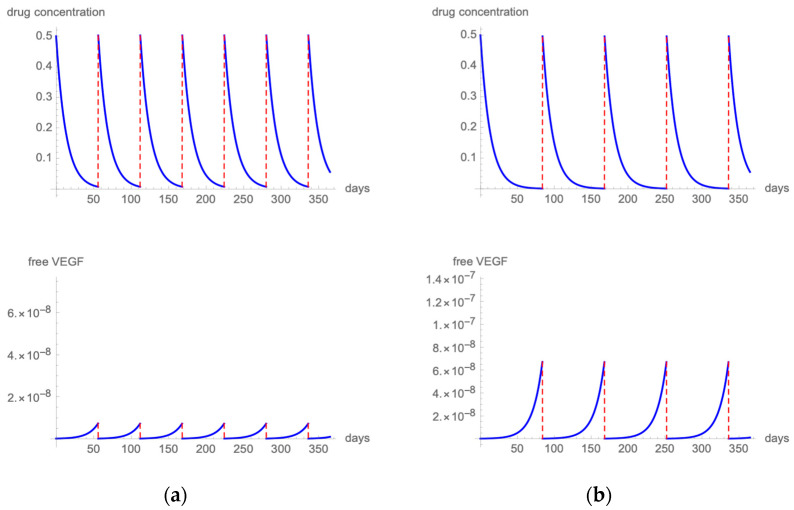
Figure 4 illustrates the intravitreal drug concentration and free VEGF proportion in a patient treated with q8 (**a**) or q12 (**b**) 2 mg aflibercept injections.

Figure 4a presents the results of the modeling of the fixed 2 mg aflibercept regimen that was evaluated in the VIEW trial [37]. The highest free VEGF levels remained below 1/10,000,000 of total VEGF. Figure 4b shows drug and VEGF kinetics when 2 mg aflibercept is administered with a q12 dosing scheme. Free VEGF spikes remain below 0.0001%.

##### Brolucizumab

**Figure 5:**
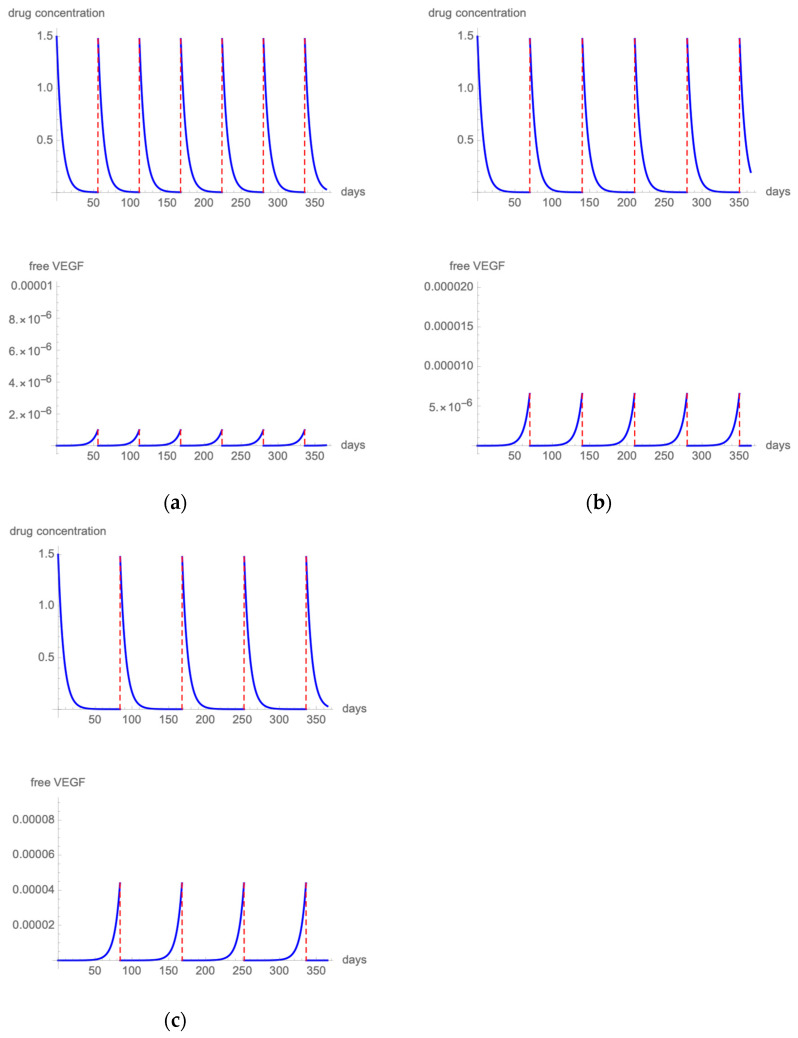
Figure 5 illustrates the intravitreal drug concentration and free VEGF proportion in a patient treated with q8 (**a**), q10 (**b**), or q12 (**c**) 6 mg brolucizumab injections.

Figure 5a presents the results of the modeling when employing a fixed q8 6 mg brolucizumab regimen [40]. The highest free VEGF levels remained at 1/1,000,000 of total VEGF. Figure 5b shows drug and VEGF kinetics when 6 mg brolucizumab is administered with a q10 dosing scheme. Free VEGF spikes remain below 0.001%. Extending the treatment interval to a q12 regimen produces spikes of free VEGF that exceed 0.004% (Figure 5c).

## 4. Discussion

Pharmacologic inhibition of intraocular VEGF is effective in the treatment of neovascular AMD, and its introduction in clinical practice has revolutionized the visual prognosis of this condition [1,2]. Optimal results were initially observed using a fixed monthly dose of 0.5 mg of ranibizumab. Unfortunately, this regimen has a demanding logistical and economic burden. To minimize these burdens, ophthalmologists soon adopted a real life-oriented treatment strategy employing a reactive, as-needed approach (PRN). In a PRN re-treatment regimen, a patient is treated only if there are signs of lesion activity. The strong point of this strategy is that the therapy is tailored to the individual patient needs, avoiding over- and under-treatment. The major downsides of PRN management are that the pathology is treated in a reactive manner, patients have to be monitored monthly, and the benefits of the treatment are highly dependent on physicians’ skill, clinic capacity, quality of diagnostic tools, and re-treatment criteria. Even in the presence of timely visits and well-defined re-treatment criteria, as in the CATT trial, still there was a 31% discrepancy between the ophthalmologist’s and the reading center’s observations when assessing OCT findings to make decisions about re-injection [42]. A large body of evidence now suggests that employing a PRN approach in a real-life setting often produces unsatisfactory results, mainly during the maintenance phase. Patients typically experience a gradual deterioration of visual function after the initial visual acuity gain obtained during the loading phase (3 monthly injections) [43]. This is chiefly attributable to an inadequate number of injections that result from logistic and clinical inefficiencies. 

A fixed regimen has the benefit of avoiding the inconsistencies resulting from variability in the application of the re-treatment criteria and can be applied in a proactive way but at the risk of over-treating some patients. For this reason, when estimating the proper inter-treatment interval time, a thorough analysis of the affinity and PK data of the anti-VEGF agent under consideration should be conducted. Accurate intraocular drug half-lives allow physicians to produce models to foresee the results of untested clinical conditions and to estimate drug efficacy periods more precisely. A reliable mathematical model can help physicians conceive better clinical studies and provide improved patient care. In light of this, we developed a mathematical model to study a series of fixed anti-VEGF treatment protocols with the aim to maintain biological efficacy while reducing healthcare burden.

Patients in the MARINA and ANCHOR trials were treated using a fixed monthly dose of 0.5 mg of ranibizumab [25,26]. Our model shows that when using a monthly treatment regimen, VEGF remains constantly blocked during the inter-treatment intervals. More precisely, the highest proportions of observed free VEGF spikes indicate that only one VEGF molecule per million was free to exert its biological activity. Clinical trial data clearly demonstrated the benefits that a fixed dose of 0.5 mg ranibizumab could offer. At month 12, patients in ANCHOR and MARINA studies experienced respectively a mean BCVA improvement of 11.3 letters and 7.2 letters from baseline. In both trials, rapid mean BCVA improvements obtained in the initial 3 months were then sustained during the follow-up, requiring monthly intravitreal injections and placing a high burden on patients and healthcare systems [25,26]. When the treatment-free interval is extended to 8 weeks, the free VEGF levels still remain under 0.001% of total VEGF, which can translate into satisfactory visual results and a reduced number of required injections, as reported by Cohen et al. [44]. Specifically, they reported a visual improvement of 8.4 letters with a treatment regimen consisting of bimonthly ranibizumab after three consecutive monthly loading doses [44]. The main advantages of this approach consist in the ability to schedule in advance all planned injections and visits while being able to maintain the initial visual gains during the follow-up. In turn, this allows a better planning of the resources needed for the management of neovascular AMD patients, improving the clinic capacity of an ophthalmology service. 

When the treatment interval is further extended to q12, 0.5 mg of ranibizumab is insufficient to keep free VEGF at low levels, and in our model, we observe free VEGF spikes that reach 0.006%. In PIER and SAILOR (naïve cohort) studies, patients received 0.5 mg ranibizumab every month for three initial injections, which is followed by quarterly dosing. This regimen led to a reduced treatment efficacy during quarterly dosing with a mean decrease of approximately five letters from month 3 to month 12 [27,45]. 

Based on data from published clinical experiences, it appears evident that an adequate treatment regimen is crucial to maintain the intraocular free VEGF below a certain threshold level, in order to maintain the initial visual gains obtained during the loading phase. Visual acuity outcomes correlate with the activity level of the neovascular lesion, which, in turn, depends on the quantity of VEGF that is free to exert its biological activity at a specific timepoint [46,47]. In our analysis, we combined and juxtaposed the clinical outcomes from various published reports and free VEGF spikes that we inferred from the model. This comparison suggests that such a free VEGF threshold level could be set at 0.001%. This inference and the results from our model have been furtherly validated by a work by Muether et al. that analyzed the temporal correlations of VEGF suppression, recurrence of neovascular AMD, and visual acuity loss following intravitreal ranibizumab [46]. The authors observed that neovascular AMD activity, as determined by spectral domain optical coherence tomography, occurred at an average of 94 days after the previous ranibizumab injection. Reactivation was never observed earlier than 8 weeks after the previous treatment.

When considering bevacizumab, our mathematical model suggests that a free VEGF proportion under the threshold level could be obtained administering 1.25 mg of bevacizumab with a fixed q4 or q8 regimen. The q4 regimen maintains the unbound VEGF level at 0.00002%, and this treatment protocol was widely investigated in the CATT trial [31]. This study reported that a fixed dose of bevacizumab administered every 4 weeks was non-inferior to a fixed monthly dosing of ranibizumab (8.0-letter gain versus 8.5-letter gain, respectively). According to our mathematical model, fixed bimonthly bevacizumab also permits maintaining the free VEGF level under 0.0001%. This treatment protocol was investigated by several authors [48,49], which reported that a q8 approach provides visual results that are non-inferior to monthly bevacizumab during a follow-up of 12 months. 

Aflibercept has a higher affinity to VEGF than other approved anti-VEGF agents. Our model shows that when aflibercept is administered bimonthly, only one VEGF molecule per 10,000,000 remains unbound. This has been clinically validated by the VIEW trials when 2-month dosing of aflibercept was as effective as monthly ranibizumab in maintaining initial BCVA gains during the follow-up [37]. Our simulation shows that if the dosing interval is increased to 12 weeks, the proportion of free VEGF still remains under 0.0001%. To date, there are no published studies evaluating aflibercept with a fixed q12 regimen. However, from ALTAIR and ARIES studies, some stimulating information could be gathered. In both trials, patients received a loading dose of three monthly injections of aflibercept followed by a treat-and-extend (TAE) regimen [50,51]. According to this proactive treatment protocol, the interval between injections can be gradually extended if anatomic and functional stability is achieved or shortened if deterioration is noted. In the ALTAIR and ARIES trials, by titrating the interval between injections on the basis of the patient’s visual and morphologic outcomes and adjusting the regimen, if necessary, the majority of patients had a mean last injection interval of at least 12 weeks and experienced a significant visual improvement at 96 weeks [50,51]. 

When considering brolucizumab, our mathematical model suggests that a free VEGF proportion under the threshold level could be obtained delivering 6 mg of brolucizumab with a fixed q8 or q10 regimen. Unfortunately, these regimens are not clinically validated, yet as in HAWK and HARRIER studies, after 3 monthly injections, eyes treated with brolucizumab received an injection every 12 weeks and were interval adjusted to every 8 weeks if disease activity was noted [40]. Brolucizumab demonstrated non-inferiority to fixed bimonthly aflibercept in BCVA change from baseline, and approximately one-half of the participants were maintained on a q12 regimen, whereas the others were switched to a q8 treatment interval during the follow-up. 

According to our mathematical simulation, when brolucizumab is administered at q12 intervals, free VEGF spikes reach 0.004%. When brolucizumab is administered at q10 intervals, free VEGF proportion remains under the threshold level of 0.001%. This latter regimen has not been clinically validated yet, but we believe it should be a candidate for a future phase IV study.

To develop the present mathematical model, some assumptions had to be made and represent the limitations of the present work. First, the affinity values used for this model were estimated using an indirect assay format that, in some circumstances, might be unsuitable for binding evaluation and affinity measures [52]. This is particularly true when testing Fab fragments. Moreover, the determination of KDa values in vivo is strongly platform-dependent and may not represent the real interaction between VEGF and anti-VEGF molecules. Second, in this model, we assumed that all anti-VEGF drugs penetrate in the same manner through the retinal layers and retinal pigment epithelium (RPE) and that the intravitreal concentrations correlate with the drug concentration at the neovascular AMDCNV level in the same way for all drugs. Third, the real, in vivo, biological KDa value could be influenced by additional factors: for example, soluble VEGF receptors that could compete with anti-VEGF inhibitors for VEGF molecules. The other limits of this model include the fact that in order to generalize the results, the main outcome was the proportion of free VEGF over total VEGF and not the actual concentration of free VEGF in a specific patient. We also estimated the threshold proportion of free VEGF that could produce optimal visual acuity results as 0.001% based on the model-generated outcomes and the comparison with actual visual acuity results from clinical studies. However, a consistent finding among all scenarios was that the best visual acuity results were obtained when the highest free VEGF spike during the first year of therapy remained under 0.001%. Finally, we only simulated the intravitreal concentrations of free VEGF-A165, but growing evidence supports the important role played in neovascular AMD activity by other angiogenic cytokines and inflammatory mediators.

Caution should be also taken in the extrapolation of these results in everyday clinical practice. Some factors may limit the applicability of the model results to real-life management of patients affected by neovascular AMD. Resistance to anti-VEGF drugs can occur at any time during the therapy (tachyphylaxis or tolerance). For instance, although VEGF is a key driver of the formation of choroidal neovascularization, many other proangiogenic factors could also promote angiogenesis, such as fibroblast growth factor, transforming growth factor, tumor necrosis factor, interleukins, platelet-derived growth factor, and placental growth factor. VEGF signaling might be closely linked to other pathways, such as platelet-derived growth factor and fibroblast growth factor signaling. An increase in the expression of these factors may possibly fuel alternate signaling pathways for angiogenesis, which could trigger VEGF-independent neovascularization and cause resistance to anti-VEGF drugs. Moreover, other drug characteristics are not taken into consideration in this mathematical model. For instance, registration trials and post-marketing studies reported an incidence of sterile intraocular inflammation in up to 4.4% of brolucizumab-treated eyes [40,53]. Some authors speculated that drug-associated inflammation, even low grade, might alter the predicted results. In addition, we studied a model that does not consider that the drug concentration in the vitreous is generally not uniform. Anti-VEGF agents are delivered to a small volume and need to spread to the boundary of the vitreous humor and to the other compartments of interest. An approach that considers the spatial aspects of the biology clearly offers advantages over compartmental models in predicting real-world outcomes. Finally, the half-lives derived from published reports are average values. At an individual level, half-lives may vary between patients, resulting in a wide range of VEGF-suppression durations within a population affected by neovascular AMD.

## 5. Conclusions

The mathematical model presented in this paper substantiates the clinical use of a fixed regimen for the treatment of neovascular AMD with ranibizumab and bevacizumab administered every 8 weeks, aflibercept every 12 weeks, and brolucizumab every 10 weeks. These treatment regimens are easily applicable in clinical practice and significantly reduce the burden of neovascular AMD management by reducing both the number of injections and monitoring visits required. These treatment protocols are worthy of interest and deserve to be further investigated in phase IV clinical studies.

## Figures and Tables

**Table 1:** Pharmacokinetic and affinity data for intravitreal anti-VEGFs.

Drug	Ranibizumab	Bevacizumab	Aflibercept	Brolucizumab
Intravitreal half-life (days)	7.19	9.82	9.1	5.1
Dose (mg)	0.5	1.25	2	6
Dissociation constant for VEGF-A165 (pM)	46	58	0.49	28.4
Molecular mass (kDa)	48	149	115	26

Legend: kDa: kilodaltons; mg: milligrams; pM: picomolar; VEGF: vascular endothelial growth factor.

## Data Availability

Not applicable.
